# Building Capacity and Advancing Regulatory Measures to Improve Food Environments in the Region of the Americas

**DOI:** 10.3390/nu16081202

**Published:** 2024-04-18

**Authors:** Carmen Levis, Carolina Mejía Toro, Sofía Rincón Gallardo Patiño, Victor Eduardo Villalobos-Daniel, Carla Spinillo, Fabio da Silva Gomes

**Affiliations:** 1 Pan American Health Organization, Washington, DC 20037, USA; villalovic@paho.org (V.E.V.-D.); gomesfabio@paho.org (F.d.S.G.); 2 NCD Alliance, 1202 Geneva, Switzerland; 3 School of Population Health, University of Auckland, Grafton, Auckland 1023, New Zealand; 4 INFORMAS (International Network for Food and Obesity/Noncommunicable Diseases, Research, Monitoring and Action Support), Grafton, Auckland 1023, New Zealand; 5 Cardiovascular Health Program, Global Health Advocacy Incubator, Washington, DC 20005, USA; srincon@advocacyincubator.org; 6 Center for Nutrition and Health Research, National Institute of Public Health, Cuernavaca 62100, Mexico; 7 Department of Design, The Federal University of Paraná, Curitiba 80060, Brazil; cgspin@gmail.com

**Keywords:** food policy, capacity building, regulatory measures, Region of the Americas, nutrition, food environments

## Abstract

Noncommunicable diseases (NCDs) are the main cause of death globally (70%) and in the Region of the Americas (80%), and poor diets are a leading driver of NCDs. In response, the Pan American Health Organization (PAHO)/World Health Organization (WHO) introduced a set of evidence-based regulatory measures to help countries improve diets through the reduced consumption of processed and ultra-processed foods. This paper aims to describe the needs of and propose actions for key actors to advance these measures. A workshop was designed to assess member states’ regulatory capacity. A thematic analysis was conducted to analyze regional needs, successes and challenges. Thereafter, the Government Capacity-Building Framework for the prevention and control of NCDs was used to examine findings. The findings were organized in two sets: (i) PAHO/WHO actions to support member states and (ii) key actors’ actions to advance regulatory policies. The results show notable regulatory progress across the Region of the Americas. However, progress differs between countries, with opportunities to strengthen measures in most countries, mainly in conflict of interest management. The results identified important actions to strengthen the regulatory capacity of PAHO/WHO member states. To maximize momentum for these actions, timelines must be identified, and political commitment can be boosted by applying human rights-based and food system-wide approaches.

## 1. Introduction

Globally, noncommunicable diseases (NCDs) and their risk factors are the main causes of morbidity, disability and mortality. NCDs cause greater than 70% of deaths worldwide and constitute one of the major public health challenges of the 21st century [1]. Specifically in the Region of the Americas, NCDs account for over 80% of all deaths, with diet-related NCDs, including ischemic heart disease and type 2 diabetes, being the leading drivers [2].

Food environments determine the type of dietary patterns people are able to follow [3]. On a global scale, the availability, affordability and marketing of ultra-processed food and drink products favor eating choices that increase the incidence and worsen outcomes of diet-related NCDs [4,5]. Ultra-processed food and drink products are industrial formulations synthesized from food substrates or prepared with substances extracted from foods or food constituents. These formulations contain little or no whole natural food; are typically energy dense and lacking in nutrients that are key to health maintenance and healthy growth; and are generally high in added sugars, saturated fat, trans fat and/or sodium (e.g., soft drinks, sweetened yogurts, sweetened breakfast cereals, convenience foods, industrially produced baked goods, ice creams or savory snacks) [5,6]. Due to their nutrient profiles, the regular intake of these products can increase the risk of developing diet-related NCDs [5,6,7,8,9,10,11,12,13,14,15,16,17,18,19,20].

Given that ultra-processed foods have market advantages over less-processed items [21], and considering their role in the development of diet-related NCDs [4,5,6,7,8,9,10,11,12,13,14,15,16,17,18,19,20], a comprehensive and coherent strategy that spans across multiple sectors is needed [22]. Evidence has shown the cost-effectiveness of implementing certain regulatory measures that help reduce the demand for and offer of processed and ultra-processed products [21,22,23]. Key technical advisory documents have been issued globally and in the Region of the Americas to support countries to adopt, implement and strengthen these regulatory measures as part of the package of efforts to prevent and control diet-related NCDs [23,24,25,26,27,28]. Based on the best evidence available of cost-effective interventions, these documents call for (a) labeling and marketing regulations of processed and ultra-processed products high in critical nutrients; (b) the taxation of sugar-sweetened beverages (SSBs) and processed and ultra-processed products high in critical nutrients; and (c) the regulation of food in school environments and other settings where food is served or offered [23,24,25,26].

One of the most cost-effective interventions to address NCD risk factors from a population-level approach is the front-of-pack labeling (FOPL) system, which provides consumers with direct, fast-capturing information to easily identify products that contain excessive amounts of critical nutrients [29,30,31,32,33,34]. Among FOPL systems, research has provided evidence that those with nutrition warning signs are more effective in informing consumers of unhealthy food and drinks and contributing to healthier purchases [32,33,35].

Moreover, taxation on SSBs and food products high in critical nutrients is recommended to modify behavioral risk factors associated with NCDs. Taxation implies a triple-fold advantage because it (1) can improve consumption choices, (2) generates revenue and (3) has the potential to reduce long-term associated healthcare costs and productivity losses [36]. Furthermore, policies that restrict the marketing of processed and ultra-processed food and drinks can help reduce the demand for and consumption of these products [37]. Additionally, strategies to improve school food environments by removing less-healthy food and drink products can support the adoption of healthy eating habits from early ages [38,39].

Despite the potential benefits of these interventions, there is strong evidence that actors within and associated with the industry of ultra-processed food and drink products often attempt to weaken, distort, delay and/or impede public policies that promote dietary health and sustainability [27,28]. To combat this growing issue, in 2012, the Sixty-Fifth World Health Assembly adopted resolution WHA65.6, which, among other items, urged member states to introduce adequate mechanisms to safeguard against potential conflicts of interest (COI) in nutrition policy development and implementation. Following this request, the World Health Organization (WHO) developed a draft approach to guide countries for this purpose [27]. Next, in 2021, after consideration of the member states, PAHO/WHO launched a roadmap for the implementation of the approach in the Region of the Americas [28].

Many countries in the Region of the Americas have been seeking to adopt sound policies, actions, laws and regulations to reduce the demand for and offer of processed and ultra-processed products, in an attempt to reshape food environments in favor of healthier eating. For example, Argentina’s Healthy Eating Law came into effect in 2022, establishing front-of-pack warning labels (FOPWLs) and regulating the marketing, promotion, sponsorship and offer of products high in critical nutrients in schools, according to PAHO/WHO’s nutrient profile model [39]; Brazil developed a tool based on PAHO/WHO’s COI tool to assist key actors in identifying and preventing COI within the scope of the National School Meal Program (PNAE) [40]; and Mexico recently amended its General Health Law to include the internet and other digital platforms (e.g., social networks) in the list of advertising platforms subject to restrictions, in addition to prohibiting the use of child-targeted elements (among others) in the advertising of prepackaged food and non-alcoholic beverages [41].

Despite notable progress in the adoption of these cost-effective measures, significant barriers are often present, adversely impacting policy development and implementation [4,42,43,44]. Barriers include the interference of commercial actors in policy-making, the lack of political commitment to advance regulations and insufficient human and financial resources [42,43,44]. Therefore, there is an urgent need to generate support for countries in building their capacity to overcome such barriers [37].

Capacity building refers to the development of knowledge, skills, commitment, structures, systems and leadership to enable effective action [27,37]. Building the capacity of the Ministry of Health, policy-makers and civil society organizations (CSOs) to safeguard decision-making processes from the interference of opposing actors, and to best prevent and manage COI, is essential to attain effective and sustainable regulatory frameworks [27,37].

Recognizing the importance of building the capacity of countries to improve food environments in the region, PAHO/WHO developed a virtual workshop from March to July 2022 that sought to provide PAHO/WHO member states with an opportunity to identify capacity needs to adopt, implement and monitor regulatory nutrition measures and discuss possible actions to address such needs. The workshop was called “Regional Capacity Building of Regulatory Measures to Prevent and Manage Diet-Related Non Communicable Diseases in the Region of the Americas” and gathered insights from Ministry of Health officials, health and food policy-makers, CSO and non-governmental organization (NGO) representatives, public health advocates, academics, health practitioners and PAHO/WHO staff from country, subregional and headquarter offices. By means of a thematic analysis of participants’ contributions, this article aims to propose an action route to advance evidenced-based regulatory measures that can improve food environments and help curb diet-related NCDs in the Region of the Americas.

## 2. Materials and Methods

### 2.1. The Workshop Design 

A peer-review process was conducted to determine the design of the virtual workshop. A technical meeting with six international capacity-building and nutrition experts was convened to refine the workshop’s concept note and agenda, including the number of days the workshop would take place for and the timing of each workshop session. Based on this process, the method of the workshop was defined: seven 150 min virtual sessions from 5 to 13 July 2022 (excluding the weekend), with actors from a variety of sectors, including Ministry of Health officials, health and food policy-makers, CSO and NGO representatives, public health advocates, such as academics, health practitioners and PAHO/WHO staff from country, sub-regional and headquarter offices. To identify potential participants to invite to the workshop, PAHO/WHO staff conducted purposeful sampling by choosing candidates based on their professional expertise. Workshop organizers sought candidates across PAHO/WHO member states whose work pertained to at least one of the five evidence-based regulatory measures to improve food systems: FOPL regulations of processed and ultra-processed products; the taxation of SSBs and processed and ultra-processed products; marketing regulations of processed and ultra-processed products; regulations on ultra-processed products in schools and other settings; and COI management and prevention. Additionally, a small number of individuals from outside the Region of the Americas were invited to participate in the workshop due to their expertise in global public health nutrition, governance and COI management. All individuals were sent an invitation to attend the workshop, which included a concept note with a registration link. Those who registered were sent a background technical paper to review and key questions to consider to support them in preparing for the workshop discussions.

**Figure 1:**
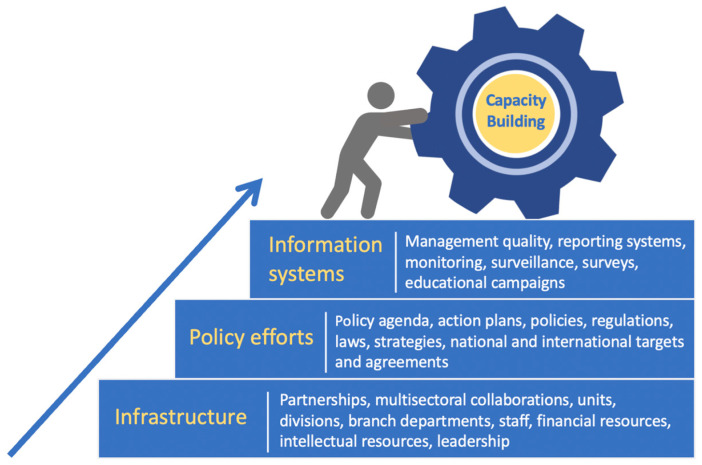
Redesigned diagram of the Government Capacity-Building Framework for the Prevention and Control of NCD proposed by Patiño et al. (2021) [38].

The workshop methodology and content were designed following the Government Capacity-Building Framework by Patiño et al. (2021) to assess national capacity for the prevention and control of NCDs [37]. The framework (Figure 1) depicts three elements of capacity building that are often used to enhance the impact and performance of public health strategies, including (1) public health infrastructure (i.e., organizational development, workforce, multisectoral collaboration and human and financial resources); (2) policy efforts (i.e., policy-making and action plans); and (3) information systems (i.e., systems to collect, monitor, report and disseminate data; surveillance and surveys). This paper also uses the Capacity-Building Framework to assess and present the findings.

### 2.2. Workshop Description

Day one of the workshop focused on discussing the capacity-building needs and opportunities to adopt, implement and monitor regulatory measures that improve food environments in the Region of the Americas. The participants then moved into randomized pre-assigned English- or Spanish-speaking breakout rooms, consisting of five to ten people to ensure a variety of sectors, and participated in a collaborative dynamic activity using Jamboard 0.2. Jamboard 0.2 is a digital platform where different persons or groups can simultaneously add information in the form of sticky notes, drawings or graphics [45]. Each group was instructed to answer questions about the capacity-building needs of their countries to adopt, implement and monitor regulatory measures that reduce the demand for and offer of processed and ultra-processed products.

**Figure 2:**
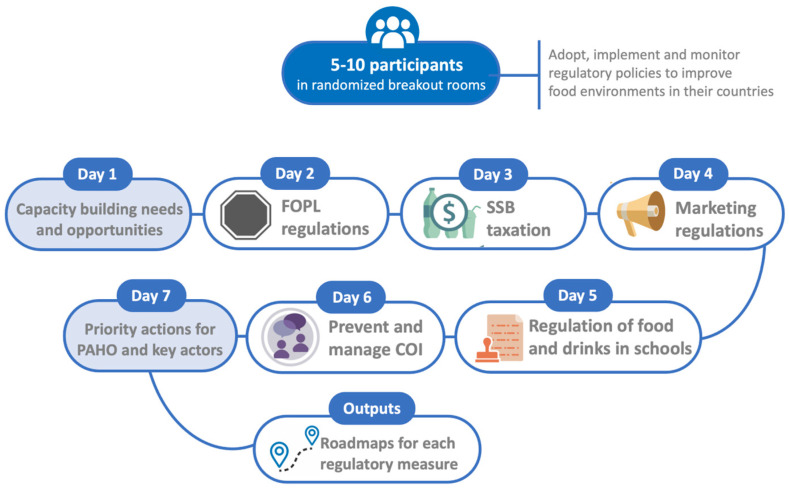
Visual synthesis of the themes discussed in the seven-day workshop.

Days two to six were each dedicated to discussing the capacity of PAHO/WHO member states to adopt, implement and monitor specific regulatory measures, with a different topic assigned to each day. Day two focused on FOPL regulations of processed and ultra-processed products; day three, on the taxation of SSBs and processed and ultra-processed products; day four, on marketing regulations of processed and ultra-processed products; day five, on regulating processed and ultra-processed products in schools and other settings; and day six, on building the capacity of countries to prevent and manage COI related to the previously mentioned regulatory measures. During the first half of the workshop sessions on days two to six, the participants were organized into randomized breakout rooms based on the primary language of the country they were representing to answer three questions via Jamboard 0.2 about the capacity-building successes, challenges and needs of their country, following the same methodology as day one (described above). During the second half of the workshop sessions on days two to six, the participants were organized into breakout rooms to develop roadmaps on capacity-building strategies for countries to adopt, implement and monitor each of the regulatory measures discussed and on preventing and managing COI. On the final day of the workshop, the capacity-building roadmaps for each regulatory measure that were developed on previous workshop days were reviewed. The participants outlined priority actions for PAHO/WHO and key actors to support member states to advance the regulatory measures discussed (Figure 2).

### 2.3. Data Analysis

Two independent researchers examined the insights collected during the seven-day workshop, conducting a thematic analysis based on the methodology proposed by Gajaweera and Johnson [46]. The analysis was carried out in several steps. To begin, the workshop discussions and content of the Jamboard 0.2 sticky notes were carefully reviewed to identify capacity needs, related concepts and possible inter-relations between concepts and to remove repeated information. Thereafter, the concepts were organized in three categories based on the Government Capacity-Building Framework: infrastructure, policy efforts and information systems [37]. Subsequently, these categories were broken into five themes concerning the main regulatory measures to improve food environments in the Americas and a sixth theme concerning capacity-building needs that cross-cut each of the matters that need regulation. A table (Table A1) was created to match the workshop contributions with their corresponding themes and categories. Using this table, contributions were analyzed, identifying patterns, divergences and convergences across contributions and themes.

Next, the roadmaps developed by the workshop participants and the stemming recommendations were analyzed. Following this analysis, Table A2 was created by consolidating key actions identified for PAHO/WHO to support member states to address their capacity-building needs to advance regulatory measures to improve food environments. These actions were organized under the three categories of the Capacity-Building Framework. Since several of the key actions intersected with multiple categories, the authors placed each action into the one category they deemed to best correspond with the action to avoid repetition.

**Figure 3:**
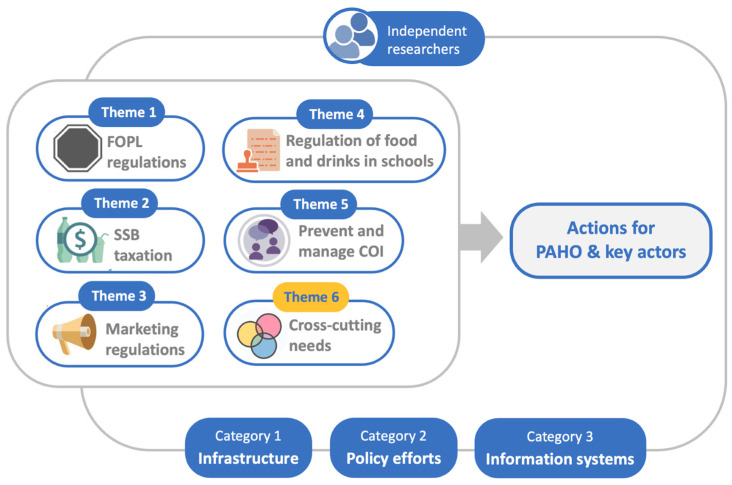
Visual synthesis of the thematic analysis of workshop results.

Next, Table A3 was created to present an action route with a set of capacity-building actions for key actors (i.e., government officials; policy-makers; CSO and NGO representatives; public health advocates, such as academics, health practitioners and PAHO/WHO staff from country, sub-regional and headquarter offices). Each actor’s set of actions was identified by establishing key themes from both the five roadmaps developed during the workshop sessions and the final workshop discussion and recommendations. The resulting themes were considered priority actions for all actors and were sorted into the three categories of the Capacity-Building Framework: infrastructure, policy efforts and information systems [37]. Table A3 also includes information on the recommended stage of each action within the five-stage Public Policy Cycle model [47,48]. The five stages of the model are (1) agenda setting, (2) policy formulation, (3) adoption (or decision-making), (4) implementation and (5) evaluation. It is worth noting that these stages rarely follow one another in linear progression, but often occur simultaneously, appear in inverse order or are rapidly skirted [47,48]. Figure 3 shows a diagram of the workshop’s overall data analysis.

## 3. Results

The virtual setting of the workshop contributed to strong attendance, resulting in a total of 126 participants (Table 1), with country representation from 27 of the 35 PAHO/WHO member states, and 5 participants who attended from countries outside of the region (Australia, Italy, Spain, the United Kingdom and Vietnam). In total, 74 of the participants worked with government organizations, 20 with PAHO/WHO, 19 with CSOs and 13 with academia.

### 3.1. Regional Capacity-Building Needs

The contributions of the workshop participants are shown in Appendix A, Table A1 regarding the regional and country capacity-building needs to adopt, implement and monitor key regulatory policies to improve food environments and prevent and manage diet-related NCDs in the Region of the Americas. Eight capacity-building needs were identified under the theme regulatory measures on FOPL and warning signs including education on the use of FOPL and related advocacy. Fifteen needs were identified under taxation on SSBs and ultra-processed foods, encompassing the need for local studies to predict the socioeconomic effects of taxation and strengthened intersectoral collaboration for tax enforcement. Ten needs emerged under the regulation of the marketing of ultra-processed food and drink products, where the need for increased civic advocacy stood out. Ten needs were obtained under regulatory measures of school food environments and other settings. In this category, one of the needs most underlined by the workshop participants was the insertion of a mandatory nutrition education course within the national school curriculum. Nineteen needs emerged under the prevention and management of COI, making this the category with the highest number of reported needs. Moreover, fourteen needs were identified as cross-cutting all of the aforementioned themes. 

### 3.2. Regional Capacity-Building Actions

The initiatives that participants proposed for PAHO/WHO to support member states to advance or strengthen the aforementioned regulatory policies were summarized into twelve capacity-building actions under the three Capacity-Building Framework categories (infrastructure, policy efforts and information systems). As shown in Appendix B, Table A2, four actions were categorized into the infrastructure category: (1) providing support for financial and human resources; (2) identifying and sharing existing member states’ government structures; (3) independent structures; and (4) hosting leadership programs. Five actions were categorized into the policy efforts category: (1) safeguarding against conflict of interest; (2) facilitating courses, workshops and webinars; (3) publishing policy guidelines and materials; (4) hosting retreats; and (5) providing technical support to member states. Lastly, three actions were identified in the information systems category: creating, managing and/or sharing: (1) observatories, (2) repositories and (3) databases.

To strengthen the capacity of PAHO/WHO member states to advance regulatory policies to help prevent diet-related NCDs in the Region of the Americas, priority actions for all relevant actors (government officials; policy-makers; CSO and NGO representatives; public health advocates, such as academics, health practitioners and PAHO/WHO staff from country, sub-regional and headquarter offices) were identified based on the roadmap results and workshop discussions. As shown in Appendix C, Table A3, the priority actions were organized into the categories of the Capacity-Building Framework, and key actors were identified for each action. Three actions were identified within the infrastructure category: (1) mapping key actors; (2) forming civil society coalitions; and (3) establishing a policy-overseeing body. Ten actions were identified within the policy efforts category: (1) creating advocacy campaigns; (2) banning industry involvement; (3) best-practice polices; (4) educational strategies for civil participation; (5) effective communication materials; (6) evidenced-based plans to face industry arguments; (7) mapping existing policies; (8) establishing legal resources; (9) identifying and mapping policy strategies; and (10) identifying and using existing resources. Six actions were identified within the information systems category: (1) obtaining country-level data; (2) developing databases; (3) evaluating policies; (4) utilizing food classification systems; (5) monitoring policy barriers; and (6) identifying monitoring systems and protocols. Furthermore, two priority challenges were identified as having the potential to hinder all of the proposed capacity-building actions: COI and financial constraints.

Although the five stages of the Public Policy Cycle model rarely follow one another in linear progression, it was determined that identifying best-practice policies, mapping existing policies, establishing policy strategies and determining an evidenced-based plan to face industry arguments are likely carried out in the agenda-setting and policy-formulation stages. Actions related to the mapping of key actors and the formation of civil society coalitions often take place during the agenda-setting, policy-formulation and implementation stages, while creating databases for processed and ultra-processed food and drink products and obtaining country-level data that identify dietary patterns and diet-related NCD rates are actions that take place during the agenda-setting, policy-formulation and evaluation stages. Monitoring systems and protocols, establishing a policy-overseeing body and evaluating policies are emphasized within the evaluation stage, while creating advocacy campaigns, banning industry from involvement in events and activities, developing effective communication materials and educational strategies for civil participation, establishing legal resources, using existing and developing new training materials and resources, monitoring policy barriers and utilizing food classification systems are actions that were identified as taking place in all stages of the policy cycle. Furthermore, it was identified that COI and limited financial resources are major challenges that could emerge in all stages of the policy process.

## 4. Discussion

This paper analyzed self-reported country needs and proposed actions for different key actors to support countries of the Region of the Americas to implement regulatory measures to improve food environments and diet-related NCDs. The results identify ten key themes for country needs; sixteen capacity-building actions for PAHO/WHO to support member states to fulfil these needs; and nineteen overall capacity-building actions for all actors.

Although capacity-building actions were identified for the region, the results indicate there are still several needs to be addressed and actions to be advanced at the country level for PAHO/WHO member states to adopt, implement and monitor the suggested regulatory measures. This is emphasized by the fact that an insufficient number of PAHO/WHO member states have implemented policies to prevent or manage NCDs: 10/35 have implemented FOPL policies (with only 8/35 using best-practice FOPWL policies) [49,50,51,52,53,54,55,56,57], 21/35 have SSB taxation policies (although most of these taxes could be further leveraged to achieve best practices and improve their impact on SSB consumption and health) [58,59] and 13/35 have food marketing regulations [35]. Furthermore, COI prevention and management to safeguard public health policies stands out as an urgent gap to address, as workshop participants reported the highest number of capacity needs under this category.

As for schools, most countries within the region have implemented at least one school food policy targeting an element of the school food environment. However, these policies are most effective when they are implemented together as part of a comprehensive food and nutrition policies approach, because this can synergistically improve the impact of all policies [60,61]. Similarly, considering food environments beyond schools are multifaceted, integrating regulatory policies that comprehensively target different aspects of these environments can also increase the overall positive impact on food environments and diet-related NCDs [62].

Most of the capacity-building needs and actions to address them that were identified during the workshop fall into the infrastructure capacity-building category [37], with a focus on two areas: augmenting human capacity and increasing funding. These results add to prior research that found an insufficient amount of human resources and infrastructure to adopt and implement diet-related regulations to address NCDs in the Region of the Americas [37]. As other studies show, investments in workforce employment and training, partnership building, research, monitoring and evaluation can effectively improve country capacities to advocate, adopt, implement and surveil policies [63,64]. Moreover, as the workshop identified, the insufficiency of human capacity can be minimized by the identification and use of pre-existing training materials and resources that help advance regulatory measures (e.g., The Virtual Campus for Public Health [65]), in addition to the use of hybrid training tools, such as the Massive Open Online Courses (MOOC), webinars and strategies like “train the trainer”.

Additionally, due to the cyclic nature of most actors and technical staff in governmental positions, it is of utmost importance to develop a sustainable system to build human capacity in order to regulate the food environment in the region. A multi-scalar approach, which incorporates inclusive planning and regular meetings to identify emerging needs and create momentum for action, can support effective collaboration on all scales, increasing human capacity in a sustainable way over time [66]. Furthermore, given that the capacity-building needs of countries and the actions to address them typically require various actors from multiple sectors (as outlined in Table A3), creating coalitions, technical advocacy and advisory groups (TAAGs) and working groups without industry interference, through the mapping of key actors who support the advancement of regulatory measures to improve food environments, could facilitate the achievement of consensus in policy development and implementation.

Another type of capacity need refers to the knowledge gap in law and jurisprudence specific to legal theories and provisions that endorse food environment regulatory initiatives. While this workshop did not explore such gaps in detail, workshop discussions revealed major knowledge gaps in this aspect across the region. First, there is a lack of clear understanding of the legal resources available at the country level that support policy development and protect food environments in defense of the human right to adequate food and nutrition, as well as children’s rights and business obligations to respect and support children’s rights in advertising and marketing. These knowledge gaps intersect with a lack of clear understanding that, first, when a risk factor is widespread in a country and affects most of the population, regulatory actions must be taken at an environmental level rather than at the individual level. Second, governments have the right and legal duty to regulate commercial activities based on arguments that support population health, and these regulations can over-ride commercial rights and international trade agreements [67]. Addressing these gaps requires the development of spaces for interdisciplinary collaboration between law and public health nutrition experts.

In this regard, across all stages of the policy cycle, COI of commercial, academic and civil society actors were identified as a common barrier to all PAHO/WHO member states to achieving the adequate implementation of unbiased, best-practice policies to improve food environments and the status of diet-related NCDs. This was an expected result as there is unequivocal evidence showing that the influence of and opposition from industries producing processed and ultra-processed food and drinks have contributed to delaying or halting the development and implementation of nutrition programs and regulatory measures that improve food environments and help prevent diet-related NCDs in many countries [43,44]. Therefore, building the human capacity to sufficiently identify, prevent and manage COI is recommended, which can be accomplished through the use of available risk assessment, disclosure and management tools that help safeguard policy development, implementation, monitoring and evaluation against possible COI, including the WHO draft approach [26] and tool [68] for the prevention and management of conflicts of interest in the policy development and implementation of nutrition programs at the country level and PAHO/WHO’s roadmap for implementing WHO’s draft approach [28]. Furthermore, mapping key actors (e.g., academia, civil society and policy-makers) can help prevent COI as it allows for the identification of coalitions, influences and interests, which supports the design of targeted mechanisms to ensure that actors who benefit from the commercialization of unhealthy commodities remain outside food/nutrition policy agenda setting and formulation [43]. In turn, this can result in strengthened transparency, partnership productivity and governance [69].

Regarding the actions proposed by the workshop participants to address the needs outlined above, specific timelines must be identified. Assigning timelines to planned policy actions is key to success in their implementation [70,71]. Empirical experience has shown that timelines can help mobilize political will, for example, through the global trans-fatty acids (TFAs) elimination strategy, where the WHO established a clear timeline with supporting tools to accelerate action towards the elimination of TFAs from the global food supply by 2023 [71]. The Public Policy Cycle stages identified for each recommended capacity-building action within Table A3 can be used to help create realistic yet flexible timelines for country action. Moreover, coordinating country timelines across the region can increase momentum, through inertia from states with more advanced regulatory performance. State actions can align on the basis of global commitments and transnational and multisectoral agendas such as the United Nations Decade of Action on Nutrition and the Sustainable Development Goals. Therefore, PAHO/WHO and key actors should consider adding timelines to the priority actions at regional, national and local levels while utilizing relevant tools to help advance policy action.

Furthermore, grounding actions by PAHO/WHO and other key actors and timelines in human rights law could help mobilize political commitment throughout all Public Policy Cycle stages [37]. For example, integrating a human rights lens during the policy agenda-setting stage could help sensitize policy-makers to the urgency of formulating, implementing and evaluating regulatory measures [37]. This is because the human rights law system has significant relevance in the legal framework of most countries in the Region of the Americas [72]. Moreover, human rights seek to protect human dignity, a principle shared across different cultures and nations [73], and adequate food and nutrition is an area of human rights inherent to human dignity and essential for the realization of other rights [74,75]. Recognizing this, most of the countries in the Region of the Americas have signed and ratified the International Covenant of Economic, Social and Cultural Rights (ICESCR) [76], which is binding to its member parties and enshrines the human right to adequate food [Art. 11(1)] and the right to the highest attainable status of health [Art. 12] [77].

### Strengths and Limitations

The main strength of this research was the data collection process. The virtual set-up of the workshop made it more accessible for actors to attend, with participation from approximately 80% of PAHO/WHO member states and eight countries outside of the Americas, including individuals from lower-funded countries. This allowed for the workshop discussions and suggestions to come from diverse country perspectives, enhancing the key learnings from the workshop and increasing the generalizability of the results. Additionally, the use of breakout rooms and activities, including the Jamboards 0.2 and roadmap templates, enabled plenty of rich discussion and information to be gathered from workshop participants, including those who were not as comfortable speaking in large groups. Furthermore, the format of the workshop—where the specific policy topics were each assigned to a different day—enabled individuals to only attend the sessions they found the most relevant to their expertise or interests. Moreover, the workshop design allowed actors to identify specific opportunities to strengthen member states’ capacity-building efforts to adopt, implement and monitor regulatory measures that, according to the best available evidence, help prevent diet-related NCDs. Finally, the workshop was also based on methodology that allowed for the collection of information in a systematic, repeatable way across countries. 

The limitations of this research relate to the theoretical framework utilized to plan and execute the workshop, the characteristics of the participant sample, the design of the workshop and the specificity of the results. Although the workshop was planned using the Capacity-Building Framework by Patiño et al. (2021), it is possible that using other human capacity-building frameworks that hold other factors constant could have yielded different results. The validity of our findings could therefore be tested in future workshops. Another limitation was that, although PAHO/WHO invited different types of actors from various sectors and different regions of the Americas, the sampling was biased towards government officials, with almost 60% of participants falling into this category. This could have resulted in unique perspectives being missed from actors working at different levels within sectors and in other areas related to the regulatory measures discussed previously. Furthermore, the seven-day workshop format with sessions lasting 2.5 h each may have led to fatigue and lower engagement for participants who attended multiple sessions. Thus, opportunities for future research include an exploration of the viewpoints and contributions of the member states and relevant sectors who were not represented during the workshop. Lastly, the results presented in this paper are specific to the Region of the Americas, which is advanced in the adoption and implementation of many of these regulatory measures. Caution should therefore be applied when applying the key actions and recommendations to other regions.

## 5. Conclusions

This research provides an initial overview of the needs and recommended capacity-building actions to support PAHO/WHO, member states and other actors to advance regulatory measures that, based on the best evidence available, curb diet-related NCDs, especially when implemented as a complimentary set of measures. The findings showed that COI prevention and management currently stands as the area of greatest capacity-building need in the Region of the Americas. Therefore, efforts must focus on safeguarding policies against COI interference in every stage of the policy cycle, from agenda setting to evaluation, so as to avoid policy delays and halts. Concurrently, national financial constraints were reported as a major capacity-building challenge for the region. Thus, key actors should seek to utilize the existing capacity-building resources available in each stage of the policy cycle to help mitigate this challenge. Prioritizing these major capacity-building needs and challenges could provide a clearer path forward for PAHO/WHO member states to develop and align regional and national regulatory strategies to reduce NCDs, including action on FOPL, taxation and marketing of processed and ultra-processed products. When these actions are implemented in a coordinated manner across sectors, through a food system- and human rights-based approach, with defined timelines, meaningful change toward improving food environments and preventing diet-related NCDs can be achieved in the Region of the Americas.

## Figures and Tables

**Table 1:** Participants’ characteristics.

Actors	Total (*n* = 126)
Government	74
PAHO/WHO	20
CSOs	19
Academia	13

Country participants attended from 27 PAHO/WHO member states, Australia, Italy, Spain, the U.K. and Vietnam.

## Data Availability

Data supporting reporting results can be found here: https://drive.google.com/drive/folders/1aUvpULZ9ULm5rA3Bsl-bb6qtt2BZPROw?usp=drive_link (accessed on 10 December 2022).

## Appendix A

**Table A1:** Thematic analysis of the capacity needs discussed during the workshop, “Regional Capacity Building of Regulatory Measures to Prevent and Manage Diet-Related Non Communicable Diseases in the Region of the Americas” (5 to 13 July 2022).

CAPACITY-BUILDING NEEDS	
Theme 1: Regulatory measures on front-of-package labeling (FOPL) and warning signs	
Category 1: Infrastructure	Formal and informal educational programs, including strategies on mass and social media to instruct end-consumers, health staff, law practitioners and governmental officers on FOPL, its importance, advocacy and mechanisms for participation in policy-making and social accountability. Create coalitions to demand evidence-based FOPL and octagonal warning signs.
Category 2: Policy efforts	Establish regulations to reduce the content capacity of packages of foods high in sugar, sodium and fat. Frontal warnings on artificially sweetened products. Binding instruments to regulate FOPL on imports. Agenda setting between neighboring countries.
Category 3: Informationsystems	Mass- and social media educational materials to orient the civil society on how to interpret FOPL and warning signs. Public regional repositories with related evidence, case examples, best practices and advocacy materials.
Theme 2: Taxation on sugar-sweetened beverages (SSBs) and ultra-processed foods	
Category 1: Infrastructure	Tax incentives and subsidies for fruit and vegetable production.
Category 2: Policy efforts	Develop reader-friendly materials explaining to policy-makers the scientific evidence concerning the economic, social and health impacts of food and beverage taxation, clarifying fears regarding any negative effects on employment rates or regressivity. Intersectoral collaboration for tax enforcement (which is still weak in many countries). Establish measures to foresee and counterbalance negative readjustment strategies by the industry (i.e., despite the tax, some industries maintain the prices prior to tax in poor areas through compensations with the marginal revenue obtained with other products or sales in other territories). Continued dialogue to align efforts between tax and health authorities at the national level and across countries of the region. Label the tax on the front of the package of the levied products.
Category 3: Information systems	Studies to foresee negative readjustment strategies of the industry and find mechanisms to counterbalance them. Reader-friendly guidelines for decision-makers on how to use and allocate the resources collected through food and beverage taxation. Forbid the sponsorship of unhealthy food and drink products by sports and wellness brands and during physical activity events. Map the barriers to tax policies. System-approach studies to measure the environmental impact of ultra-processed food and drink products. Model studies to estimate the impact of including an environmental-footprint tax (i.e., based on plastic use, water, gas emissions or eutrophication footprint). Social marketing materials to address cultural habits of the regular consumption of sugar-sweetened beverages. Information campaigns to explain and promote the mechanisms by which civil society can monitor the transparency in the collection and use of tax revenues and lobbying by the private sector. Regional information systems to exchange country taxation learning, research and experiences. Impact assessment on the role of the Ministry of Finance in monitoring tax collection and utilization.
Theme 3: Regulation of the marketing of ultra-processed food and drink products	
Category 1: Infrastructure	Resources to halt industry strategies to convey that the regulatory measures and taxes will drive economic relapse (i.e., through massive job loss, domestic gross product decrease) and/or food shortages. More law and advocacy experts familiar with food and food marketing regulation, including the demand and enforceability of the rights to food and health. Guidelines for the preparation and sale of foods and beverages in schools, based on the PAHO/WHO nutrient profile model and the NOM-051-SCFI-1994—General labeling specifications for prepackaged foods and non-alcoholic beverages.
Category 2: Policy efforts	Ban the use of cartoons and celebrities in the marketing and labeling of ultra-processed products. Need for defined, strict sanctions for violations of the regulatory measures. Need to broaden the scope of the regulatory measures not only to tackle marketing addressed to children but to include all audiences. Mandatory availability of free water dispensers in food services and public spaces. Subsidies to produce sustainably packaged water.
Category 3: Information systems	Educational campaigns designed by population target, addressing children, youths, caregivers and civil society and informing about unhealthy food marketing strategies and inviting them to report uncompliant publicity and marketing messages. These campaigns should be created by marketing experts to be as eye-catching as the industry campaigns. Educational strategies to instruct end-users on healthy, affordable food and lifestyle choices.
Theme 4: Regulation of food and drinks in schools	
Category 1: Infrastructure	Increase the offer of affordable fruits and vegetables in educational and collective settings (e.g., libraries, offices, entertainment facilities). Mandate that every educational and work setting offers free drinkable water. Establish a team of public servants to monitor regulatory compliance in educational institutions, as schools refuse to take this responsibility due to insufficient human resources and technical capacity. Incentives for schools and environments that comply with regulations.
Category 2: Policy efforts	Binding list of healthy products allowed to be sold in educational, sport and work environments. The list must establish a maximum content of calories per g/mL, sugar, saturated fats and salt and exclude all products containing artificial sweeteners and trans fats. Make malnutrition prevention programs mandatory for all schools. Align the regulations on food environments with the national dietary guidelines.
Category 3: Information systems	Workshops and easy-to-read materials clarifying industry arguments and school concerns that food-environment regulations will cause budget shortfalls to schools and school cafeterias. Studies and scientific communication materials measuring the efficiency of food-environment regulations in reducing malnutrition rates. An epidemiology unit to assess changes in the nutritional status of children in schools after regulatory implementation.
Theme 5: Prevention and management of conflicts of interest (COI)	
Category 1: Infrastructure	Form coalitions that include civil society and support them to surveil for and report incidents of COI. Divulge the “make it make sense” campaign of the Healthy Caribbean Coalition as a case example of best practices. Educational strategies in negotiation and assertive mediation addressed to advocates. Prepare universities to train students and staff in COI prevention and management. Compulsory course on COI management for high schools and universities. Strategies to strengthen the participation of non-industry multisectoral stakeholders to outnumber the representatives of the industry of unhealthy commodities. Map existing food, health and nutrition regulations to identify and denounce COI.
Category 2: Policy efforts	Establish a unique official national and regional code of conduct for COI management and prevention. National and subnational agendas specific for COI prevention and management. Binding instruments to regulate the interference of the industry through recruiting academics to publish content that contradicts the benefits of regulations on FOPL, the taxation of unhealthy products, the marketing of unhealthy foods and drinks and food environments. Reader-friendly, evidence-based resources for decision-makers and the public clarifying distorted arguments from the industry that taxes on ultra-processed products will result in unemployment, food shortages and national economic relapse. Ensure mechanisms to maintain the strategies for COI management regardless of the rotation of government officials. Legal resources to protect public officers, technicians and scholars from intimidation. Prohibit beverage and ultra-processed food companies from sponsoring sport-, education- and health-related activities and infrastructure. Regulations and legal resources to blind the design and adoption stages of a policy from private intervention, allowing the private sector to participate only until the public consultation stage. Binding regulation that defines and limits the level of industry involvement in dietary and health guidelines, programs and educational content.
Category 3: Informationsystems	Educational resources to help decision-makers and other stakeholders understand what COI are, their potential impact and how to recognize, foresee and manage them. Awareness-raising media campaigns. Evidence-based resources to address the extended perception that COI are a “difficult” matter. Map decision-makers, decision influencers and health champions to identify existing and potential COI.
Theme 6: Cross-cutting Needs *	
Category 1: Infrastructure	Human resources to (1) raise evidence-based awareness on the benefits of the regulations and taxation; (2) design, implement, monitor, adjust and evaluate policies, plans and programs; (3) oversee tax collection, allocation and use; (4) communicate the progress and findings to the community; (5) lead the educational strategies; (6) work in advocacy. Funding for national research on the impact and benefits of tax regulations. Funding and research incentives to produce national evidence in support of the regulations. Use this evidence to advocate before the legislative branch and industry lobbyists. Funding to inform research findings at local, national and regional levels. Funding to implement and monitor regulations and programs. Gather government and international cooperation funding. Regional and national platforms to synergize regulation at the regional and national levels and align the actions of the stakeholders, institutions and countries involved. The industry should not take part in the design phase of the regulations. Establish an independent body to lead this platform. Partnerships between healthcare personnel, NGO advocates, academics, grassroots organizations, multilateral organizations and government agencies.
Category 2: Policy efforts	The regulatory instruments to improve nutrition at the country level must be binding. Aligning the regulations across countries to ensure synergy at the regional level. Adopting a binding regional covenant to improve nutrition. Establish national plans to orient the industry on how to reformulate its portfolio to align with the regulations. Establish independent national and regional bodies that monitor the implementation and compliance of the regulations.
Category 3: Information systems	Educational strategies and short reader-friendly materials: (I) for decision-makers, institutional staff and civil society to learn about the importance, planning, steps to implementation and monitoring of regulatory measures to improve nutrition; (II) to improve civil society’s understanding of policy, legislation and pathways for political incidence; (III) to empower civil society to engage in policy-making and monitoring; (V) to equip professionals from areas outside health with tools to participate in health-related advocacy and policy. Information systems to educate the civic society to participate in food, nutrition and health decision-making.

* NOTE: The “cross-cutting needs” theme corresponds to all the needs referred to by the workshop participants that apply to all the themes previously analyzed (Theme 1, regulatory measures on front-of-package labeling (FOPL) and warning signs; Theme 2, taxation on sugar-sweetened beverages (SSBs) and unhealthy foods; Theme 3, regulation of the marketing of unhealthy food and drink products; Theme 4, regulatory measures of school food environments and other settings; Theme 5: prevention and management of conflicts of interest).

## Appendix B

**Table A2:** PAHO/WHO capacity-building actions to improve nutrition in the Region of the Americas.

CAPACITY-BUILDING FRAMEWORK CATEGORIES	
Category 1: Infrastructure	Financial and human resources To increase capacity to advance regulatory measures. Government structures Identify and coordinate existing government structures at all levels (national, provincial and local) which can support the formulation, adoption, implementation, monitoring and evaluation of regulatory measures. Independent structure Assist the member states in the creation of a national independent body composed of law and public health experts to map food, health and nutrition regulations; identify loopholes; advise the legislative branch to fill the gaps; monitor compliance and denounce violations. Leadership programs Provide a leadership program where regional leaders are identified and learn by experience about a given topic for a year term.
Category 2: Policy efforts	Conflict of interests Establish unified international parameters that guide the national codes of conduct to prevent and manage conflicts of interest. Courses, workshops and webinars Develop online or in-person workshops and virtual webinars on topics such as intersectoral cooperation; sensitizing ministries to these policies; and the sharing of policy success stories and good practices from various countries. Develop educational courses, such as the capacity-building course PAHO/WHO will be launching in 2023, which focuses on each of the regulatory measures highlighted in this paper. Policy guidelines and materials Reader-friendly, evidenced-based guidelines and tools that can help policy-makers draft legislation. Guidelines to help health authorities, advocates and civil society to identify and counterbalance industry interferences in regulatory drafting, implementation, monitoring and evaluation. Retreats Dedicated to a specific regulatory measure (e.g., FOPL, the taxation of ultra-processed products, COI management, etc.). Technical support Meet with member states to provide technical support on (among other topics): Drafting regulations and action plans, based on evidence and best practices. Coupling regulations on food environments with regulations on other public spaces and with other norms on food labeling, advertisement and taxation. Devising a strategy to help schools implement new regulations. Assisting the ministries of education and health within the region to design and implement a compulsory subject on healthy, sustainable living in the national education plan.
Category 3: Information systems	Observatories Facilitating collaboration between academia, civil society and governments. Track and advance the regulatory agenda discussed in this workshop. Coordinate nutrition regulations with strategies beyond food-related policies, such as tobacco- and alcohol-related policies. Repositories Share existing repositories: RIS is consolidated as the digital library of PAHO/WHO. Examples of external repositories include the World Public Health Nutrition Association (WPHNA) (international) and the Latin American and Caribbean Nutrition and Health Community of Practice’s (COLANSA’s) group repository (Latin America and the Caribbean). Databases Create and share databases with materials addressed to civil society, advocates, private, public and nonprofit organizations.

## Appendix C

**Table A3:** Capacity-building actions for all actors to improve nutrition in the Region of the Americas, with actors’ roles and stages of the Public Policy Cycle identified for each capacity-building action.

CAPACITY-BUILDING FRAMEWORK CATEGORIES		
**Capacity-Building Actions**		**Actors’ Roles ***
Category 1: Infrastructure	Key actors and connections ^1,2,4^ Map key policy actors (e.g., government, policy-makers, civil society, academics) and opposers throughout the policy process specific to each of the regulatory measures. Identify key members from other sectors to strengthen the internal integration of the institutions involved in the implementation of the regulation(s). Map the decision-makers and actors with influential power that support or might support COI regulation. Map the non-food/nutrition industry actors that can be affected by or interested in nutrition, food and health regulation and the links and alliances between them. Make the maps publicly available. Promote the maps as a resource to support actors to make informed choices when partnering with industry actors or when buying from them. Civil society ^1,2,4^ Create coalitions or working groups without industry interference to develop harmonized strategies across sectors. Ensure that sufficient budget is allocated to strengthen civil society initiatives.	Government officials, PAHO/WHO, CSOs, NGOs, academia ______________________________ Government officials, PAHO/WHO, CSOs, NGOs, academia ___________________________ Government officials, PAHO, CSOs, NGOs, academia ___________________________ Government officials, PAHO/WHO, CSOs, NGOs, academia ___________________________ Government officials, PAHO/WHO ___________________________ Government officials, PAHO/WHO, CSOs, NGOs ___________________________ Government officials, CSOs, NGOs ___________________________ Government officials, PAHO/WHO, CSOs
Category 2: Policy efforts	Bans ^1,2,3,4,5^ Ban industries of ultra-processed products from funding or sponsoring educational and sporting events, nutrition and health activities. Best practices ^1,2^ Identify global regulatory policies to prevent diet-related NCDs with the highest standards that protect health over commercial interests. Seize momentum of political opportunity by adopting a regulatory measure when other countries are also adopting or implementing it or by proposing a bill while another one is in progress (i.e., Chile and Peru could have negotiated the package of regulations on food labeling, advertisement and school environments all at once). Educational strategies for civil participation ^1,2,3,4,5^ Formal and informal education strategies addressed to the general public to instruct civil society on the use of the constitutional mechanisms to engage in policy-making and monitoring. Formal and informal education strategies addressed to the general public to instruct on how to monitor advertisements and denounce violations of ad hoc regulations. Effective communication materials ^1,2,3,4,5^ Work with marketing experts to create promotional campaigns using appropriate language for the intended audience. Plan and design campaigns in mass and digital media. Evidenced-based plans to face industry arguments ^1,2^ Evidence-based plans that civil society can adopt to its context to combat communications and publications by industry claiming that regulatory and tax measures on the labeling, expenditure and advertisement of ultra- processed foods and beverages will drive to loss of job sources, domestic food shortages and economic relapse. Map existing policies ^1,2^ Identify and map existing regulatory measures that are being discussed and are implemented nationally and regionally to understand where to prioritize work. Identify regulations beyond the ones focused on ultra-processed products regulatory policies (e.g., tobacco and alcohol policies) to find key learnings that can be transferred over to promote policy coherence and increase engagement across sectors. Legal Resources ^1,2,3,4,5^ Establish resources for legal defense to combat industry arguments regarding intellectual property (a recurrent type of argument to limit emerging marketing restrictions). Define legal protections from misleading advertisements. Establish legal instruments to protect public officers, technicians and scholars from being threatened, intimidated or dismissed when they refuse to yield to private interests. Policy strategies ^1,2^ Track regulatory agendas and strategies that have already been developed and implemented beyond food-related policies and across sectors, to learn from successful experiences and failures, such as tobacco and alcohol. Develop specific binding frameworks that define the stages of the policy cycle at which the private sector can and cannot participate and establish monetary and non-monetary heavy sanctions for violations. Establish measures to regulate marketing addressed to both children and all audiences ^6^. Establish regulations that cover all mass media including YouTube and social media. Establish regional, cross-border regulatory frameworks for food labeling, marketing and advertisement. Harmonize and synergize the regulations on food taxation, labeling, marketing, school environments and other settings. Training and resources ^1,2,3,4,5^ Identify and use existing tools to strengthen actors’ capacity to support regulatory measures (e.g., PAHO/WHO’s nutrient profile model, the REPLACE TRANS FAT online course and PAHO/WHO’s publications such as FOPL as a Policy Tool for the Prevention of NCD). Develop continuing education materials and courses as needed to train students, policy-makers and practitioners in (I) food nutrient profiles; (II) choices for affordable healthy lifestyles; (III) how to monitor food environments for regulatory compliance; (IV) how to denounce irregularities.	Government officials ___________________________ Government officials, PAHO/WHO, CSOs, NGOs, academia ___________________________ Government officials, public health advocates ___________________________ Government officials, CSOs, public health advocates ___________________________ Government officials, CSOs, public health advocates ___________________________ Government officials, PAHO/WHO, CSOs ___________________________ Government officials, PAHO/WHO, CSOs ___________________________ Government officials, PAHO/WHO, CSOs, academia ___________________________ Government officials, PAHO/WHO, CSOs ___________________________ Government officials, PAHO/WHO, academia ___________________________ Government officials, PAHO/WHO, academia ___________________________ Government officials, PAHO/WHO ___________________________ Government officials, PAHO/WHO ___________________________ Government officials, PAHO/WHO, academia ___________________________ Government officials, PAHO/WHO ___________________________ Government officials, PAHO/WHO ___________________________ Government officials, PAHO/WHO ___________________________ Government officials, PAHO/WHO ___________________________ Government officials, PAHO/WHO ___________________________ Government officials, PAHO/WHO, public health advocates ___________________________ Government officials, CSOs
Category 3: Information Systems	Country level data ^1,2,5^ Design and disseminate national surveys with dietary patterns and health indicators to estimate the economic costs of ultra-processed product consumption. When a lack of country data exist, data from other countries could be used. Databases ^1,2,5^ Identify and/or develop up-to-date national, sub-regional and regional databases on regulatory measures on ultra-processed food and drink products. Evaluation ^5^ To establish a system to assess the results of the implementation of the regulatory measures. Food classification systems ^1,2,3,4,5^ Utilize food guidelines, nutrient profile models and classification systems in tools for governments to identify processed and ultra-processed products. Monitor policy barriers ^1,2,3,4,5^ To monitor and map existing policy barriers that delay or halt the advancement of the regulations in order to plan targeted strategies. Monitor systems and protocols ^5^ Identify monitoring systems and protocols (e.g., INFORMAS) that have science-based frameworks and indicators in place that could be replicated across policies and countries and could be compared within the region over time. Create entities in charge and/or delegate to existing entities with adequate safeguards for the prevention of COI.	Government officials, PAHO/WHO ___________________________ PAHO/WHO, government officials, CSOs ___________________________ Government officials, PAHO/WHO, academia ___________________________ Government officials, PAHO/WHO, CSO ___________________________ Government officials, PAHO/WHO, CSOs, academia ___________________________ PAHO/WHO, CSOs, academia ___________________________ Government officials, NGOs, CSOs
**Priority challenges that can hinder all actions**		
	Conflicts of interest ^1,2,3,4,5^ Industry interference in all stages of the policy process due to financial power and other sources of power. Financial constraints ^1,2,3,4,5^ A common barrier for many countries, so it is important for countries to have an awareness of the existing capacity-building tools available in order to maximize and utilize pre-existing resources. For example, some countries already have national product labeling databases that could be utilized by other countries that have similar markets as an option to move forward through the policy process.	

******* Actors’ Roles: For each capacity-building action, the key actors that are commonly involved in that work were listed. However, depending on the specific country and context, the actors involved may vary. ^1^ Agenda setting: This step refers to the process through which a policy and the problem it is intended to address are acknowledged to be of public interest. ^2^ Formulation: In this step, the public administration concerned examines the various policy options it considers to be possible solutions. ^3^ Adoption: Adoption is the stage during which decisions are made at the governmental level, resulting in a decision that favors one or more approaches to addressing a given problem. ^4^ Implementation: At this stage, the policy’s implementation parameters are established, which can directly affect the eventual outcome of the policy. ^5^ Evaluation: This is the stage during which a policy is evaluated, to verify whether its implementation and its effects are aligned with the objectives that were explicitly or implicitly set out (NCCHPP, 2013). ^6^ To avoid the country-to-country differences in the legal age to define children or minors. These differences create legal gaps that can be used by the industry to advertise unhealthy commodities.
